# The Birmingham Hospital Saturday Fund

**Published:** 1896-09-26

**Authors:** 


### THE BIRMINGHAM HOSPITAL SATURDAY FUND.

It is often said that there is no smoke without a fire, and notwithstanding the disclaimer inserted by Mr. Smedley in the *Birmingham Daily Post* in regard to our criticism of his scheme for celebrating the silver wedding of the Hospital Saturday, we cannot accept the statement there made that the scheme which we so criticised existed only in our imagination. We are glad to find from Mr. Smedley's letter that the smoke which we detected in Hospital Saturday affairs indicated only a small blaze, and that that has been promptly quenched, but that there was an intention to utilise the Hospital Saturday organisation for purposes which have nothing whatever to do with the hospitals or with any legitimate purpose connected with the fund, we cannot but believe—unless, indeed, Mr. Smedley has been much less perspicuous in his statements to his interviewer than he usually is to those with whom he speaks. The scheme as it *now* stands appears to be merely a project for erecting a building in which various societies and movements connected with the working men of the town shall be able to have their offices, if they choose so to do, and in the large hall of which the Hospital Saturday meetings shall be held. Mr. Smedley says in the *Daily Post*, "The extent of any connection with the Hospital Saturday Fund is this—that suitable accommodation shall be provided for its work and meetings, and that at someone else's cost." We are glad to hear it, and, if this pronouncement is the result of our comments, glad indeed that we criticised the project as we did. Let us turn back to the *Birmingham Daily Argus* of the week before, in which the interview referred to appears. This paper says that the author of "this big building scheme" is no other than "the indefatigable organiser of the Hospital Saturday movement, whose busy and fertile brain has for some months been occupied with yet another important development of the great cause with which he is so closely identified." Again, "Said Mr. Smedley: 'I want to make the Hospital Saturday movement the basis of a big work in the town. The organisation is too strong and too good not to do more work,'" and then he goes on to describe some of the manifold objects embraced in his scheme—mutual aid societies, an employment bureau, sick societies, a large provident dispensary, a high-class social club—all these were embraced in the "big work" of which Mr. Smedley wanted "to make the Hospital Saturday movement the basis." It was stated that the scheme was "all-comprehensive and cosmopolitan. 'Why should we not do something with this great organisation?' said Mr. Smedley with enthusiasm. 'There is no city in the country or in the world where such a splendid organisation exists. The men are ready to work, and trained to work, and there is no limit to its future possibilities.'" We cannot but think that anyone who pays Mr. Smedley the compliment of attributing to his words their proper meaning will agree that in the scheme so put forward there was enough to justify us in a protest against using the organisation of the Birmingham Hospital Saturday for the furtherance of projects "having nothing whatever to do with the prime object of the Hospital Saturday movement." The matter lies between Mr. Smedley and his interviewer; but we think that the smoke they raised between them was good evidence of a fire somewhere, which boded no good to the Hospital Saturday Fund, and we are glad to be assured that it has been put out.

